# Flavones and Flavone Glycosides from* Salvia macrosiphon* Boiss

**Published:** 2011

**Authors:** Ahmad Reza Gohari, Hakimeh Ebrahimi, Soodabeh Saeidnia, Mahdi Foruzani, Puneh Ebrahimi, Yousef Ajani

**Affiliations:** a *Medicinal Plants Research Center, Faculty of Pharmacy, Tehran University of Medical Sciences, Tehran, Iran.*; b *Department of Chemistry, Faculty of Sciences, Payame Noor University, Sari, Iran, *; c *Gonbad Institute of Higher Education, Gonbad, Iran. *; d *Institute of Medicinal Plants, ACECR, Tehran, Iran.*

**Keywords:** Lamiaceae, *Salvia macrosiphon*, Flavonoids, Steroid

## Abstract

*Salvia* genus, which is generally called Maryam-Goli in the Persian language, belongs to Lamiaceae family and comprises 58 species in Iran. Four flavonoids plus a steroid compound were isolated from the ethyl acetate and methanol extracts of the aerial parts of *Salvia macrosiphon *Boiss, using different chromatographic methods on the silica gel and sephadex LH_20_. The structures of the isolated compounds were determined to be apigenin-7, 4’-dimethyl ether (1), *β*-sitosterol (2), salvigenin (3) apigenin-7-O-glucoside (4) and luteolin-7-O-glucoside (5) using the ^1^H, ^13^C-NMR and MS spectra in comparison with those reported in the literature.

## Introduction

*Salvia* genus, which is generally called Maryami or Maryam-Goli in the persian language, belongs to Lamiaceae family and includes around 58 species in Iran (1). Among them *S. sahendica*,* S. urmiensis*,* S. persepolitana* and *S. hypoleuca* excursively grow in Iran and the others grow in iraq, armenia, egypt, russia, saudi arabia and anatoly (1-3). Previous phytochemical investigations revealed the presence of phenolic acids and polyphenols, flavonoid glycosides, anthocyanins, diterpenes and sesquiterpenes in the several S*alvia* species (4, 5). *Salvia* has been used in traditional and folk medicine as a diuretic agent, tonic, anti rheumatoid and chronic pains, antimicrobial, carminative, flavor and spices since antiquity (4).

*Salvia macrosiphon* Boiss. is a quite-abundant and polymorphic plant in Iran and afghanistan. It is a perennial, herbaceous, strongly aromatic, lemon-scented and pale yellowish green plant. Its stems are few to several from a woody rootstock, up to 60 cm, erect, sturdy, quadrangular, below eglandular pilose, above with a dense indumentum of short glandular hairs and sessile oil globules (1). Till now, we have studied the genetic relations among some *Salvia* species using molecular biological assays which showed that* S. macrosiphon *and *S. Aethiopis *are extremely alike and *S. brachyantha *has a genetic distance far from *Satureja *species (6).

Literature reviews show that there are few reports on phytochemical investigation of *S. macrosiphon*. Recently, composition of the essential oil of *S. macrosiphon* has been investigated by GLC and GC-MS. Sixty-four components, representing 93.3% of the oil, were characterized. The main constituents of the oil were reported as linalool (26.3%), hexyl hexanoate (9.6%), hexyl isovalerate (9.3%), hexyl-2-methyl-butanoate (8.9%), sclareol (7.2%) and hexyl octanoate (6.1%) (7).

Previously, the chemical composition of the essential oil of *S. macrosiphon*, collected around Tehran, was reported (8). The main components of the oil of *S. macrosiphon* were *α*- gurjunene (11%), *β*-cubebene (10.6%) and germacrene-B (7%). Furthermore, isolation and identification of some flavonoids (salvigenin, eupatorin and 13-epi-manoyl oxide) has been reported from the aerial parts of *S. macrosiphon* originated of Lorestan Province (9). 

In this paper, we aimed to report the separation and structural elucidation of the main phytochemical constituents from the aerial parts of *S. macrosiphon* which has not been previously reported. 

## Experimental

*Plant’s material*

Aerial parts of *S. macrosiphon*, at the full flowering stage, were gathered around Damavand on the way of Tehran-Firuzkouh road (June, 2008). A voucher specimen (6674- THE) of the plant deposited at the Herbarium of the Faculty of Pharmacy, Medicinal Plant Research Center, Tehran University of Medical Sciences. Plant specimen was identified by Dr. Gholam Reza Amin. 

*Instruments and materials*

The^ 1^H and ^13^C-NMR spectra were measured on a Brucker Avance TM 500 DRX (500 MHz for ^1^H and 125 MHz for ^13^C) spectrometer with tetramethylsilane as an internal standard and chemical shifts were given in δ (ppm). The MS data were recorded on an Agilent Technology (HP TM) instrument with 5973 Network Mass Selective Detector (MS model). The silica gel 60F_254 _pre-coated plates (Merck TM) were used for TLC. The spots were detected by spraying anisaldehyde-H_2_SO_4_ reagent followed by heating (120°C for 5 min).

*Isolation process*

The flowered aerial parts of *S. macrosiphon* (960 g) were cut into small pieces and percolate with ethyl acetate and methanol, consequently, at room temperature. The ethyl acetate extract (77 g) was subjected to silica gel column chromatography (CC) with hexane : CHCl3(9 : 1, 5 : 5, 0 : 1), CHCl_3 _: AcOEt (5 : 5) and AcOEt as eluent to give six fractions (A-F). The fraction C (135 mg) was submitted to sephadex LH_20_ CC with methanol as an eluent to obtain five fractions C_1_-C_5_. The fraction C5 (31 mg) was chromatographed again on sephadex LH20 to result in compound 1 (15 mg). The fraction C2 (195 mg) was subjected to silica gel CC with hexane : AcOEt (8 : 2 and 0 : 1) to gain six fractions (C21-C26). The fractions C22 and C25 were compound 2 (8 mg) and 3 (2 mg), respectively.

The MeOH extract (60 g) was successively subjected to silica gel column chromatography and washed with AcOEt and MeOH as eluents to result in 2 main fractions M1 (8 g) and M2 (45 g), respectively. About one half of M2 (20 g) was fractionated on silica gel CC with CHCl3 : MeOH (8 : 2, 6 : 4, 4 : 6 and 0 : 1) to obtain 7 fractions M11 –M17. The fraction M12 (315 mg) was purified twice on sephadex LH20 with MeOH to yield compound 4 (13 mg). The fraction M14 (810 mg) was chromatographed on sephadex LH20 with MeOH to afford Compound 5 (9 mg).

## Results and Discussion

**Figure 1:**
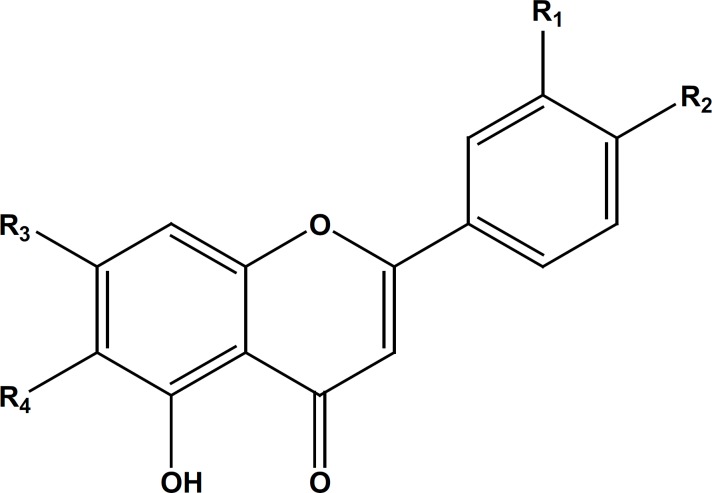
The structures of the isolated flavonoids from *Salvia macrosiphon*.

Isolated compounds (Figure 1) from the ethyl acetate and MeOH extracts of S. macrosiphon identified as, apigenin-7, 4’-dimethyl ether (1), *β*-sitosterol (2), salvigenin (3) apigenin-7-O-glucoside (4) and luteolin-7-O-glucoside (5) by comparing their NMR and MS spectral data with those reported in literature (10-13). Tables (1 and 2) show the results of the ^1^H and ^13^C-NMR for the isolated flavonoids 1, 4 and 5. 

1 R_1_ = H ; R_2_ = OCH_3_ ; R_3_ = OCH_3_ ; R_4_ = H

3 R_1_ = H ; R_2_ = OCH_3_ ; R_3_ = OCH_3_ ; R_4_ = OCH_3_

4 R_1_ = H ; R_2_ = OH ; R_3_ = OGlc ; R_4_ = H

5 R_1_ = OH ; R_2_ = OH ; R_3_ = OGlc ; R_4_ = H

**Table 1:** The ^13^C-NMR of the flavonoid components isolated from *Salvia macrosiphon*

**Carbon No.**	**1** ^a^	**4** ^ b^	**5** ^ b^
1	-	-	-
2	163.9	164.4	164.4
3	104.3	103.0	99.8
4	182.4	181.8	181.8
5	157.6	145.7	162.1
6	98.0	99.4	95.3
7	165.3	150.9	162.9
8	92.5	94.6	95.6
9	162.5	149.9	156.9
10	105.0	105.3	103.1
1’	123.5	121.0	121.3
2’	128.0	128.1	113.5
3’	114.4	115.9	145.7
4’	164.0	161.3	149.9
5’	114.4	115.9	115.9
6’	128.0	128.1	119.0
1’’	-	99.8	99.4
2’’	-	73.1	73.0
3’’	-	76.5	76.3
4’’	-	69.5	69.5
5’’	-	77.1	77.1
6’’	-	60.5	60.5
7-OMe	55.7	-	-
4’- OMe	55.5	-	-
6-OMe	-	-	-

Note: a measured in CDC_l3_, b in DMSO-d_6_.

**Table 2:** The ^1^H-NMR of the flavonoid components isolated from *Salvia macrosiphon*

**Carbon No.**	**1** ^a^	**4** ^ b^	**5** ^ b^
1	-	-	-
2	-	-	-
3	6.54 (*s*)	6.88 (*s*)	6.72 (*s*)
4	-	-	-
5	-	-	-
6	6.49 (*d*, *J *= 2.5)	6.84 (*d, J *= 1.8)	6.83 (*d, J *= 1.8 )
7	-	-	-
8	6.37 (*d, J *= 2.5)	6.94 (*d, J *= 1.8)	6.78 (*d, J *= 1.8)
9	-	-	-
10	-	-	-
1’	-	-	-
2’	7.85 (*d, J *= 9)	7.96 (*d, J *= 8.7)	7.41(*d, J *= 1.8)
3’	7.02 (*d, J *= 9)	7.96 (*d, J *= 8.8)	
4’			
5’	7.02 (*d, J *= 9)	7.19 (*d, J *= 8.8)	6.88 (*d, J *= 8.3)
6’	7.85 (*d, J *= 9)	8.04 (*d, J *= 8.7)	7.44 (*dd, J *= 8.5, 1.8)
1’’	-	5.07 (*d, J *= 7.3)	5.07 (*d, J *= 7.3)
2’’- 6’’	-	3.17-3.49 (*m*)	3.16-3.73 (*m*)
6-OMe	-	-	-
7-OMe	3.89 (*s*)	-	-
4’- OMe	3.88 (*s*)	-	-
5-OH	12.8	12.9	12.5

Note: a measured in CDC_l3_, b in DMSO-d_6_

The compound 1 gave the characteristic 1H-NMR spectrum possessing the same substitution pattern of apigenin rings: A (5, 7 disubstituted), B (4’ monosubstituted). Both proton and carbon spectrum showed the presence of two methyl ether group substituted at 7 and 4’ positions (Tables 1 and 2). The proton signal of 5-OH could be detected at 12.8 ppm, therefore, the compound 1 is identified as apigenin-7, 4’-dimethyl ether (14, 15). The compound 4 indicated the aromatic proton and carbon chemical shifts of the apigenin glycoside. The presence of one glucosyl moiety with characteristic signals at 5.07 ppm (d, *J* = 7.3) for anomeric proton and 3.17-3.49 ppm (H-2’’ to H-6’’) was confirmed. The glucose moiety was substituted at 7 – O position based on ^13^C-NMR data compared to references (14).

The flavone salvigenin (3) was assigned by comparison of its NMR data with those reported in references (12). The pattern of B ring in salvigenin was similar to compound 1 but there was a different pattern in the A ring (5 hydroxy and 6, 7 dimethoxy). The chemical shifts of the carbon signals in salvigenin represented three methyl ether group substituted at 6, 7 and 4’ positions. In relation to the compound 5, the glucose moiety must be connected to 7-O position of the flavon, luteolin. The compound 5 was identified as luteolin-7-O-glucoside, by comparing the 1H and ^13^C-NMR spectra with published data (10, 11). 

Among the flavones and glycosides isolated from S. macrosiphon, the compounds apigenin-7, 4’-dimethyl ether (1), apigenin-7-O-glucoside (4) and luteolin-7-O-glucoside (5) are reported for the first time from this plant. The effect of apigenin-7-glucoside (A7G) on skin inflammation, induced by different generators of reactive oxygen species and free radicals, has been studied. The results indicated the inhibition of skin inflammation by administration of A7G in a dose dependent manner (16). 

Recently, we reported the presence of luteolin-7-O-glucoside (L7G) in *Dracocephalum* species from Lamiaceae family (10, 14). So far, it is reported that L7G significantly inhibited the PDGF-BB-induced (platelet-derived growth factor-BB) proliferation and the DNA synthesis of the VSMCs (vascular smooth muscle cells) in a concentration-dependent manner (17). It seems that the abnormal proliferation of aortic VSMCs plays an important role in the pathogenesis of atherosclerosis and also in the development of hypertension (17-19). Anti-asthmatic activity of L7G (isolated from *Ailanthus altissima*) was evaluated in an* in-vivo* murine asthmatic model and the results suggested that the anti-asthmatic activity of L7G in ovalbumin-induced lung inflammation may occur in part via the down regulation of T-helper 2 cytokine transcripts as well as the inhibition of prostaglandin E_2_ production (20). Apigenin-7, 4’-dimethyl ether was previously isolated as the major compound of *Teucrium polium* and showed antioxidative activity (15). 

In conclusion, many plant families are particularly rich in the flavone compounds, and one of them is the Lamiaceae (Labiatae) that show a large variability of structures. Flavonoids in *Salvia *species have such a potential, not only to indicate the biological and pharmacological activities, but also to provide the useful taxonomic characters, especially at the infra specific level (to distinguish populations from different geographic origin), and possibly at the specific and sectional levels (21, 22).
